# Validity and responsiveness of the Clubfoot Assessment Protocol (CAP). A methodological study

**DOI:** 10.1186/1471-2474-7-28

**Published:** 2006-03-15

**Authors:** Hanneke Andriesse, Ewa M Roos, Gunnar Hägglund, Gun-Britt Jarnlo

**Affiliations:** 1 Department of Orthopedics, Clinical Sciences, Lund University and Lund University Hospital, SE-221 85 Lund, Sweden; 2 Departments of Health Sciences, Division of Physical Therapy, Lund University, Lasarettsgatan 7, SE 221 85, Sweden

## Abstract

**Background:**

The Clubfoot Assessment Protocol (CAP) is a multi dimensional instrument designed for longitudinal follow up of the clubfoot deformity during growth. Item reliability has shown to be sufficient. In this article the CAP's validity and responsiveness is studied using the Dimeglio classification scoring as a gold standard.

**Methods:**

Thirty-two children with 45 congenital clubfeet were assessed prospectively and consecutively at ages of new-born, one, two, four months and two years of age.

For convergent/divergent construct validity the Spearman's correlation coefficients were calculated. Discriminate validity was evaluated by studying the scores in bilateral clubfeet. The floor-ceiling effects at baseline (untreated clubfeet) and at two years of age (treated clubfeet) were evaluated. Responsiveness was evaluated by using effect sizes (ES) and by calculating if significant changes (Wilcoxons signed test) had occurred between the different measurement occasions.

**Results:**

High to moderate significant correlation were found between CAP **mobility **I and **morphology **and the Dimeglio scores (r_s _= 0.77 and 0.44 respectively). Low correlation was found between CAP **muscle function**, **mobility II **and **motion quality **and the Dimeglio scoring system (r_s _= 0.20, 0.09 and 0.06 respectively). Of 13 children with bilateral clubfeet, 11 showed different CAP **mobility I **scores between right and left foot at baseline (untreated) compared with 5 with the Dimeglio score. At the other assessment occasions the CAP **mobility I **continued to show higher discrimination ability than the Dimeglio. No floor effects and low ceiling effects were found in the untreated clubfeet for both instruments. High ceiling effects were found in the CAP for the treated children and low for the Dimeglio.

Responsiveness was good. ES from untreated to treated ranged from 0.80 to 4.35 for the CAP subgroups and was 4.68 for the Dimeglio. The first four treatment months, the CAP **mobility I **had generally higher ES compared with the Dimeglio.

**Conclusion:**

The Clubfoot Assessment Protocol shows in this study good validity and responsiveness. The CAP is more responsive when severity ranges between mild – moderate to severe, while the Dimeglio focuses more on the extremes. The ability to discriminate between different mobility status of the right and left foot in bilaterally affected children in this population was higher compared with the Dimeglio score implicating a better sensitivity for the CAP.

## Background

Studies on children with clubfoot are mainly based on classification and cross-sectional group studies with often a wide range in age. Consequently most instruments used in clubfoot assessment are primarily for classification or cross sectional long-term outcome. There is a lack of knowledge on the nature of clubfoot progression. Therefore the development of an instrument for longitudinal multilevel follow-up is of importance.

As a part of the ongoing study of the Clubfoot Assessment Protocol [1] psychometric properties, the purpose of this study was to investigate validity and responsiveness of the CAP in comparison with the Dimeglio scoring system [2].

Research questions were: *i*. How does the CAP subgroups associate with the Dimeglio instrument? *ii*. How are the ceiling -and floor effects in these two instruments and how is the sensitivity for levels of different severity? *iii*. Do these instruments show change over time?

## Methods

### Subjects and treatment program

Thirty-two consecutive children diagnosed with congenital clubfoot (19 unilateral, 13 bilateral = 45 clubfeet) were assessed prospectively as new-borns (at the time of presentation to the orthopaedist), at 1 and 2 months (preoperative), at 4 months (postoperative), and at 2 years of age with the CAP [1] and the Dimeglio classification system [2]. The assessments were done in the clinic in conjunction with patient's treatment and did not involve extra inconvenience for the child or the parents. All assessments were done by an experienced physiotherapist (HA). The Ethics Committee at Lund University Hospital approved the study. All Subjects gave their informed consent to participate.

Treatment was started within 8 days after birth in all cases. The patient group received one of two treatment regimes during the first 4 months. Sixteen children were treated according to a modified Copenhagen method [3] and 16 children were treated using only the first part of the Ponseti method [4] namely the casting technique regime but not the Foot Abduction Orthosis (FAO).

**Figure 1:**
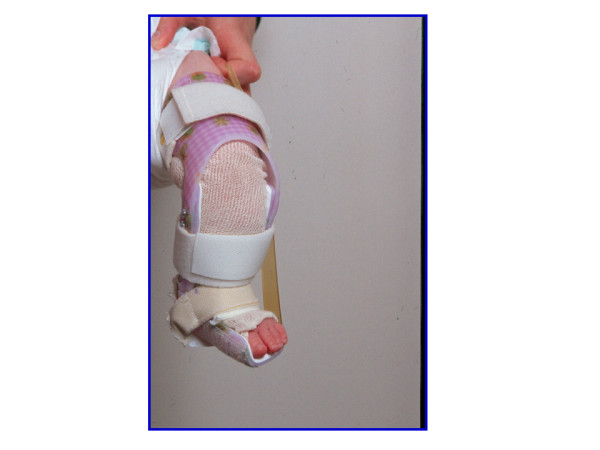
Dynamic Knee Ankle Foot Orthosis (with permission from Scandinavian Orthopaedic technical Laboratory).

In both treatment regimes operation was done at the age of two months. Operation criteria for surgery was the same in both groups; A remaining isolated equinus position of less than 5° dorsiflexion was treated with percutaneous achilles tendon lengthening, if necessary in combination with posterior capsulotomy. The varus – adductus (inversion) component being less than 15° mobile into valgus-abduktion (eversion) was treated with tibialis posterior lengthening and capsulotomy of the talo-navicular joint. Remaining toe-flexion was treated with lengthening of flexor hallucis and/or flexor digitorum tendon. Treatment goal was a foot with at least 15° dorsiflexion and more than 15° of eversion. In this population 16 feet (35%) required an achilles tenotomy alone, 20 feet (44 %) had achilles tenotomy and a posteriomedial release, one foot had achilles tenotomy and elongation of flexor hallucis longus and 8 feet (18%) in five children had no operative intervention. Postoperatively, or directly at two month of age if no operation was needed, the children continued their treatment with an individual made dynamic Knee Ankle Foot Orthosis (KAFO, Figure 1). The foot part was positioned in outward rotation, related to the obtained eversion of the foot (varying between 15°–35°). The dynamic construction (hinged ankle joint and elastic band) of the KAFO makes it possible to keep the achilles tendon lightly stretched during the night. The elastic band was kept lose during daytime enabling the foot to move freely in plantar – and dorsal flexion. This orthosis was used according to a standardised schedule during the first 2 months (at least 18 hours a day; whole night and maximal 2 hours free morning time, afternoon and before bedtime). During free time muscle function in foot and knee movement was stimulated by play-full activities stimulating especially the peroneus longus muscles. Gradually the orthose usage during daytime was cut down. At the age of eight month all children only use the orthose(s) night time (12 hours) and during midday nap. Once the child started to walk independently the KAFO was changed to a dynamic Ankle Foot Orthosis (AFO). Foot outward rotation was maintained around 10 to 15°. Compliance was set to a minimal 10 hours a night. This is continued until the age of four years. Generally no orthopaedic shoes were prescribed.

### The Clubfoot Assessment Protocol (CAP)

In a previous study we have described the Clubfoot Assessment Protocol (CAP) and its item reliability which was found to be moderate to very good [1]. The same study showed that the instrument was able to show variation on impairment and activity level in different phases of the treatment.

After this study we have slightly revised the protocol. Multi-correlation analyses have shown (unpublished data) that it was possible to exclude two items, tightness and squatting, without decreasing information on the clubfoot child's clinical function. The scale construct for domain motion quality was revised from 4 to 5 levels. Feedback from other clinical users on this part of the instruments showed the need for specifying an extra response item between "cannot "and "deviant" (Table 1).

The concept behind the construct of the CAP was that within orthopaedics firstly the mobility is of major interest (i.e. correction of the deformity), secondly, the muscle function for control over the foot which influences its development, thirdly, the exterior of the foot plays an important roll for patient satisfaction and finally, activity which is a combination of mobility, muscle function and neuro-motoric development and of importance for patient satisfaction and participation. Twenty items divided between Body structure (CAP subgroups; mobility I and II, muscle function and morphology) and Activity (CAP subgroup; motion quality) levels according to the International Classification of Function, Disability and Health (ICF) [5] form the CAP. The CAP is intended to be used in clinical practice and research, in short- and long term follow-up during the child's growth. Focus is on item and subgroup level and no total scores are used. Scoring intervals for each item are determined by their expected impact (importance) on activity and clinical decision making and normal variation. Item scoring range from 0–4 (worst to best).

### Dimeglio classification scoring system

We chose the Dimeglio classification system for comparison [2] (Table 2) as it is one of the most cited instruments and is used both for classification and in follow-up studies [6-12].

This instrument assesses primarily the mobility of the clubfoot deformity and is comparable with the first 5 items in the subgroup mobility of the CAP. One item concerns muscle function.

The Dimeglio classification consists of 8 items. Scorings for 4 items range from 0–4 (best to worst). Four items only score zero or one. Total score ranges between 20-0 (very severe: 16–20, severe 11–15, moderate 6–10, and postural 0–5). Focus is on total score and classification. Good reliability has been shown [2,6,13].

To simplify comparison between these two instruments the raw scores of both instruments were transformed into percentage scores (0–100, worst to best possible score).

The differences between these two instruments are primarily that the Dimeglio scoring focuses mostly on mobility. Secondly the width of the scoring ranges is different with the CAP focusing its scoring more to the centre and less to the extremes.

**Figure 2:**
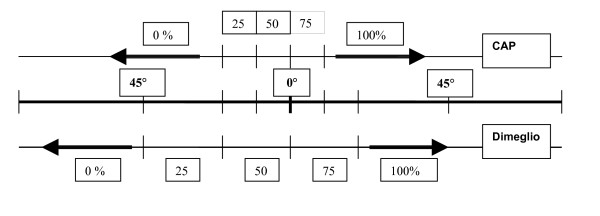
Proportion in scoring distribution between the Dimeglio scoring system and the CAP for the item "derotation/eversion".

An example of the item derotation/inversion-eversion is given in figure 2.

### Validity

#### Convergent/divergent construct validity

This was determined by assessing the relationship between the CAP and the Dimeglio score in the new-born phase and at 2 years of age. We expected a high correlation (convergent validity) between the CAP **mobility I **and the Dimeglio score as they mainly measure the same construct i.e. mobility. A moderate correlation was expected with the CAP morphology, as we assume that morphology is influenced by the mobility. Also moderate correlation was expected between CAP **motion **quality and the Dimeglio score as mobility is a prerequisite for functional ability. Low correlation was expected with the CAP **mobility II **and CAP **muscle function as **muscle testing (length and strength) is a different construct than mobility (divergent construct validity).

The Spearman correlation coefficient (*r*_*s*_) for non-parametric data was used. P < 0.05.

*The floor and ceiling effects *for the CAP and the Dimeglio score were assessed at two occasions; at baseline/new-born (untreated) and at the age of 2 years (treated).

#### Discriminant validity

The ability to show variation (that is being sensitive for difference) is one of the aims of the CAP. Score intervals were chosen less broad in the middle of the CAP **mobility I **compared with the Dimeglio scoring system. These two instruments ability for showing variation was assessed by comparing their ability to differ in severity between the right and left foot in the 13 bilateral clubfeet. The right and left foot were compared at new-born, preoperatively and at two years of age.

### Responsiveness (= longitudinal construct validity)

The CAP and the Dimeglio assessments were applied at age new-born (the pre-treatment phase), 1 month, 2 (pre-operative), 4 months (post-operative) and at 2 years of age.

Responsiveness was calculated for both instruments by use of effect size (ES) [14]. Effect size was defined as the mean change scores divided by the standard deviation of the baseline score, which in this case is the score in new-born. Effect sizes of 0.2 are defined as small, 0.5 as medium and 0.8 as large [15].

Finally we assessed if changes had occurred across the whole follow up period with Friedman's tests for change. Thereafter change between a measurement and its preceding assessment was assessed by using Wilcoxon's signed rank test and the Friedman's test for change across the whole follow up period. P < 0.05 was considered significant.

The SPSS 12.00 was used for statistical analyses.

## Results

### Missing data

Item "M. soleus-gastrocnemius." in CAP **muscle function **could be tested properly in less than 40 % of the children. Therefore this item was distracted from this subgroup and omitted from further analysis.

There were 14 CAP subgroup scores missing from a total of 945 assessments (= 1.5 %) at five time points. At one month two scores for morphology were missing. Preoperatively, two scores were missing for **mobility II**, four for **morphology **and six for muscle function. None were missing for the Dimeglio score (Table 3).

### Validity

#### Convergent/divergent construct validity

The highest correlation was found between the CAP **mobility I **and the Dimeglio score (r_s _= 0.77) (Table 4). Moderate correlation was seen with the CAP **morphology**. No correlation was found with the CAP **motion**. (r_s _= 0.06), the CAP **muscle function **nor the CAP **mobility II **(r_s _= 0.2 respectively 0.09).

#### Ceiling and floor effect

No floor effects were seen for the CAP or for the Dimeglio scoring at the two assessment occasions (Table 5). At new-born (untreated clubfeet) 37% ceiling scores were found for subgroup CAP **mobility II **and 4 % for CAP **muscle function **indicating muscle length and strength within normal variation. At two years of age (treated clubfeet) all CAP subgroups had highly increased ceiling effects ranging from 36 to 87%. Subgroup CAPmotion showed at age 2 years a ceiling effect of 38 % indicating that 15 out of 45 feet had walking and running patterns within normal variation. The Dimeglios scores showed nearly no ceiling effects at all (4 %).

#### Discriminant validity

Of the 13 children with bilateral clubfeet, 11 showed different scores between the right and left foot when the CAP **mobility I **was used, compared to 5/13 when the Dimeglios scoring was used. In the preoperative assessment 10/13 showed variation for the CAP and 4/13 for the Dimeglio. At two years of age this was 4/13 for CAP and 0/13 for the Dimeglio.

### Responsiveness

All children except two out of 32 were compliant with the orthosis treatment. All CAP subgroups showed effect sizes varying from medium to high (range 0.60 to 4.35, Table 6). The CAP **mobility I **and the Dimeglio score showed generally the highest effects sizes with a slight tendency to higher efficiency of the CAP **mobility I **which in the later treatment phase is passed by the Dimeglio.

Highest effects sizes were found during the first month of treatment (Table 6). The CAP subgroups showed effect sizes from 0.60 to 2.70. The Dimeglio scoring showed 2.42. Increase of the effect sizes slowed down between one to two months of treatment, increasing strikingly for the CAP **mobility I **and Dimeglio score from pre-to postoperative. Thereafter the feet continued to improve in mobility and muscle function with a clearly higher effect size for the Dimeglio score (4.68) compared with the CAP **mobility I **(4.35) at the age of two years. The CAP **mobility II **and **morphology **showed lower ES at the age of two years (0.80 and 1.66 respectively) compared with the age of four month (1.00 and 2.01 respectively) implying deterioration.

Both instruments showed significant changes between the scoring occasions except between the pre-and postoperative measurement for the CAP **mobility II**, **muscle function **and **morphology **and between postoperative to 2 years of age for the CAP **mobility I **and **mobility II **(Table 3). From baseline (= newborn) to the age of two years all the CAP subgroups scores and the Dimeglio scoring showed significant (Friedman's test, p < 0.0001) improvement.

## Discussion

Within the field of clubfoot assessment a countless amount of instruments are available. A search on databases (Medline, Libris and Elin) on methodological aspects in clubfoot measurements revealed seven instruments on clubfoot with documented reliability studies [16-21]. All of them were developed primarily for classification except for Roye et al. [20] which is used as a patient based outcome instrument. Content/face validity was based on expert groups deciding a gold standard. No validity studies were found, except for Roye et al. One study, comparing three clubfoot measurements, regarded responsiveness [7] by utilising the Wilcoxon signed rank test.

Furthermore, within paediatric orthopaedics, different measurements on activity and participation levels and methodological sound are now available depending on diagnose and aim of the study [22-24]. For studies on clubfoot these instruments might not be sensitive enough as these children are normally high functioning [19].

The results of this study on methodological aspects of a clubfoot assessments instrument shows that the associations between the CAP and the Dimeglio scoring were generally in accordance to the presumptions. Both CAP and the Dimeglio score showed god ability to detect change over time though the CAP showed higher sensitivity for discriminating differences than the Dimeglio score. The distributions of the scores were in accordance with the instruments aim and scale construct. The CAP adds other clinical dimensions on impairment and activity level compared with the Dimeglio score which mainly assesses mobility.

### Validity

#### Convergent and divergent construct validity

The high to moderate correlation between the CAP subgroups **mobility I **and **morphology **and the Dimeglio indicates a clear association between these domains. The correlations seen between the CAP subgroups **mobility **II and **muscle function **were low and indicate that different entities are assessed. The low correlation found between the CAP **motion quality **and the Dimeglio was not expected. As the CAP **mobility I **correlated so well with the Dimeglio score we checked the correlation of the Dimeglio score with the CAP domain motion quality and found a highly significant moderate correlation (0.41, p = 0.01). It seems that the scale construct of the CAP **mobility I **give assessments that correlates better with the activity levels of the clubfoot child than the Dimeglio score.

#### Ceiling and floor-effect

Normally, the high ceiling effects seen in the treated group with the CAP would be found to be a negative factor in measurements as these effects make it impossible to measure improvement. The scoring construct of the CAP though concentrates on smaller intervals in the middle of the scale, where changes are of most clinical importance. The CAP is not intended to measure changes above normal or below extreme abnormal.

In the untreated group of clubfeet children both instruments showed no floor effects in this population. Moderate ceiling effects where found for CAP **mobility II **(37%) which indicates that about 60% of the children have problems with this item, which is of clinical importance. In the follow-up, at two years of age most of the children should have reached a functional level within normal variation. As the CAP has its anchor points within normal variation and the Dimeglio score has its anchors on more extreme levels (e.g. forefoot abduction > 20° or valgus >20°), the CAP will sooner reach its ceiling levels. Furthermore with usage of only three scoring levels such as in domain CAP **muscle function**, there is less room for discrimination which gives higher ceiling- or floor effects.

We conclude that both the CAP and the Dimeglio floor and ceiling effects are in accordance with the concept and construct of the instruments.

#### Disciminant validity

As a result of different scaling intervals and distribution of scores, the use of different instruments can result in different conclusions. This can be seen in the different abilities of these two instruments sensitivity when both instruments assess the same patient group. Our clinical experience is that children with bilateral clubfeet most often have a difference in severity between left-right. The results of this study shows that if we used the Dimeglio score we would have concluded that most children with bilateral clubfeet had similar severity. The CAP **mobility I **though showed that out of 13 children with bilateral clubfeet 11 showed different scores between left-right. In this case one can conclude that the commonly seen differences in severity in bilateral clubfeet can be assessed and both feet can thus be included in clubfeet studies.

### Responsiveness

The result on responsiveness showed for both instruments large effects sizes. The effect sizes of the CAP domains should not be compared with each other as they assess different entities.

The CAP **mobility I **shows higher effect size compared with the Dimeglio when the feet are still clinically in a worse state. This is maintained until the postoperative phase where the Dimeglio effect size increases between 4 month and age 2 years compared with the CAP **mobility I **(with1.10 to 4.68 and 0.26 to 4.35, respectively). This is caused by the fact that the best possible score is reached earlier with the CAP **mobility I **than the Dimeglio. Furthermore both instruments contain slight different items such as plantar flexion for the CAP **mobility I **and integration of muscle function in the Dimeglio score, increasing or decreasing the subgroup score.

We tried to compare our study with Lehman et al. [7] which showed to be problematic as baseline groups differ in both severity and age and treatment programs were not totally comparable.

The effect sizes for the CAP subgroups **mobility II**, **muscle function **and **morphology **ranged from medium (0.60) to very large (2.1), increasing with time during treatment the first 4 months. In phase two of treatment (maintaining correction) muscle function continues to develop though the length of the toe flexors and morphology seem to decrease.

It is interesting that these CAP subgroups show changes over time providing new information from different dimensions. This will enable us to gain more specific information and make outcome evaluation on different levels possible. For example we can, by looking at the development of the subgroup muscle function, conclude that once the feet have increased mobility, muscle function slowly improves. Subgroup morphology shows a decrease at the age of two years. We analysed the results on item level which showed that decreased scoring of the item tibia rotation was the main cause. This is an example of the effects of aggregating information into a single total score. We notice a decrease, but cannot distinguish the cause, as the other items in this subgroup regard other problems which are not associated with tibia rotation even though they belong to this subgroup. This could be an effect of low item-internal consistency for this domain (calculated by Crohnbachs alfa and normally used in testing reliability in patient based questionnaires [25]). That is why it is also important to observe how the individual items develop. The same accounts for studies on group or individual level.

Even though the instruments show good responsiveness on group level through statistically detectable change it is more and more emphasised to study responsiveness in relation to the patient/parents perceived clinical important change [26,27]. Future studies will therefore be needed.

### Methodological issues

In this material none of the children's clubfeet were assessed as extremely severe which would probably have resulted in a higher differentiating ability with the Dimeglio score.

Item "M. soleus-gastrocnemius" seems to be difficult to assess properly in this young age group as co-operation and understanding the meaning of the test is low. We suggest that this item should not be expected to be assessed before the age of four years.

We had problems to find a methodological soundly developed clubfoot instrument to compare our newly developed Clubfoot Assessment Protocol (CAP) with. The Dimeglio scoring system seemed to be the best alternative. When we started this study the Outcome Evaluation in Clubfoot [28] generated by Bensahel et al. and the International Clubfoot Study Group (ICSG) and now advocated as one of the instruments to be used for outcome measures was not available. The construct of the ICSG- instrument has some similarities with the CAP and in future it will be interesting to see more studies on these instruments validity, sensitivity and responsiveness.

## Conclusion

The Clubfoot Assessment Protocol with its different subgroups shows in this study good validity and responsiveness. The CAP is more responsive regarding severity in the moderate to severe range, while the Dimeglio focuses more on the extremes. The ability to discriminate between different mobility status of the right and left foot in bilaterally affected children in this population was higher compared to the Dimeglio score. The CAP provides information on the development of different functional domains within clubfoot diagnosis. As validation and development of an instrument is a complicated procedure more studies will be needed to fully establish the use of the Clubfoot Assessment Protocol.

## Competing interests

The author(s) declare that they have no competing interests.

## Authors' contributions

HA and GH designed and collected the data. HA and EMR analysed, interpreted and revised the data and manuscript. HA drafted the manuscript. GH and G-BJ revised the manuscript.

## Pre-publication history

The pre-publication history for this paper can be accessed here:



## Figures and Tables

**Table 1:** The Clubfoot Assessment Protocol (CAP version 1.1).

Name:	Date of birth:				
Date of assessment:	Assessment number:				
Side:	O Left	O Right			
Rating	**0**	**1**	**2**	**3**	**4**
**Passive mobility**					
**I.**					
1. Dorsiflexion	<-10°	-10°-<0°	0°-<+10°	+10°-+20°	>+20°
2. Plantar flexion	0°-<10°	10°-<20°	20°-<30°	30°–40°	>40°
3. Varus/valgus	>20°var	20°-<10°var	10°-<0°var	0°-neutral	>0°vlg
4. Inv/evers	>20°inv	20°-<10°inv	10°-<0°inv	0°–10°evrs	>10°ev
5. Add/abd	>20°add	20°-<10°add	10°-<0°add	0°-neutral	>0°abd
					
**II.**					
6. Flx.dig.long.	+ reduced		reduced		normal
7. Flx.dig.hall.	+ reduced		reduced		normal
					
**Muscle function (strength)**					
8. M. peroneus	absent/poor		reduced		normal
9. M. ext.dig.long	absent/poor		reduced		normal
10. M. sol./gastr.	absent/poor		reduced		normal
					
**Morphology**					
11. Tibial rotation	+ inw.		inw.		normal
12. Calcaneus position	>10 varus		>0 varus <10		neutral/vlg
13. Forefoot position	>20° add.		>10 add. <20°		add<10°
14. Foot arch	+ cavus/planus		cavus/planus		normal
					
**Motion quality**					
**I.**					
15. Running 2 y	cannot	+deviant	deviant	slightly deviant	normal
16. Walking 2 y	cannot	+deviant	deviant	slightly deviant	normal
17. Toe walking 3 y	cannot	+deviant	deviant	slightly deviant	normal
18. Heel walking 3 y	cannot	+deviant	deviant	slightly deviant	normal
					
**II.**					
19. One legstand 4 y	cannot	+deviant	deviant	slightly deviant	normal
20. Hop1leg 4 y	cannot	+deviant	deviant	slightly deviant	normal
Extra notes: Structured questions about pain, stiffness, shoe problems, physical condition, activity level, sports and social participation and patient/parent satisfaction.					

+ = Pronounced/very, var = varus, vlg = valgus, inv = inversion, evers = eversion, add = adduction, abd = abduction., inw = inward rotation, flx.dig.long. = length of M. flexor digiti longus, flx.dig.hall. = length of M. flexor digiti hallucis, y = years.

^©^Hanneke Andriesse 2005

**Table 2:** Dimeglio classification scoring system

**Rating**	**4**	**3**	**2**	**1**	**0**
1. Equinus	45–90°pltf	20°–45°pltf	20°pltf – 0°	0° – 20°dorsx	>+20°dorsx
2. Varus	45–90var	20°–45°var	20°var-0°	0°–20°vlg	>20°vlg
3. Supination	45–90sup	20°–45sup	20°sup-0°	0°–20°pron	>20°pron
4. Adductus	45–90°add	20°–45°add	20°add – 0	0°>-<20 abd	>20°abd
5. Posterior crease				yes	no
6. Medial crease				yes	no
7. Cavus				yes	no
8. Deviant muscle function				yes	no

pltf = plantarflexion, dorsx = dorsalflexion, var = varus, vlg = valgus, sup = supination, pron = pronation, add = adduction, abd = abduction.

**Table 3:** Mean (SD) in percentage score for the CAP and Dimeglio scores. 0–100 worst to best possible score.

	New-born	1 month	2 months, preoperative	4 months, postoperative	Age 2 years
**CAP**					
Mobility I	43.20 (11.83)	75.00 (13.65)**	80.10 (11.30)**	93.44 (4.80)**	94.67 (5.05)
Mobility II	73.90 (23.20)	87.80 (17.37)**	93.60 (15.50)*	97.78 (7.20)	92.22 (20.52)
Musclefunction	52.80 (20.80)	79.40 (18.70)*	90.38 (18.68)*	91.70 (16.01)	96.70 (8.60)*
Morphology	62.40 (16.60)	87.50 (13.40)**	94.81 (6.80)**	95.80 (9.20)	90.00 (10.20)**
Motion					82.80 (18.13)
**Dimeglio**	49.70 (8.50)	70.20 (9.00)**	73.70 (8.50)**	83.70 (5.20)**	89.44 (3.34)*

* p < 0.05, ** p < 0.001 Wilcoxon's signed rank test calculated from one occasion to the next follow-up occasion.

**Table 4:** Convergent and Divergent Construct Validity. The Spearman's correlation coefficient (r_s_) was used. N = 45.

**CAP**	Expected correlation	Dimeglio-newborn (p value)
Mobility I	High	0.77 (0.000)
Mobility II	Low	0.09 (0.55)
Musclefunction	Low	0.20 (0.20)
Morphology	Moderate	0.44 (0.002)
Motion	Moderate	0.06 (0.72) a

a = assessed at age 2 years

**Table 5:** Ceiling and floor effects in percentage of patients assessed with the CAP and the Dimeglio instrument at two treatment phases, untreated and treated clubfeet. N = 45

	Floor/Ceiling New-born	Floor/Ceiling 2 years
**CAP**		
Mobility I	0/0	0/35
Mobility II	0/37	0/84
Muscle function	0/4	0/86
Morphology	0/0	0/42
Motion	-	0/38
**Dimeglio**	0/0	0/4

**Table 6:** Effect Size from baseline to 1 month, 2 month, 4 month and 2 years of treatment for all clubfeet (n = 45), starting treatment within 8 days after birth.

	Effect Size 1 month	Effect Size 2 months, preoperative	Effect Size 4 month, postoperative	Effect Size, 2 years
**CAP**				
Mobility I	2.70	3.2	4.21	4.35
Mobility II	0.60	0.80	1.00	0.80
Musclefunction	1.28	1.60	1.87	2.1
Morphology	1.55	1.84	2.01	1.66
				
**Dimeglio**	2.42	2.82	4.00	4.68
